# Association Rule Learning Is an Easy and Efficient Method for Identifying Profiles of Traumas and Stressors that Predict Psychopathology in Disaster Survivors: The Example of Sri Lanka

**DOI:** 10.3390/ijerph17082850

**Published:** 2020-04-21

**Authors:** Nuwan Jayawickreme, Ehsan Atefi, Eranda Jayawickreme, Jiale Qin, Amir H. Gandomi

**Affiliations:** 1 Department of Psychology, Manhattan College, Bronx, NY 18966, USA; 2 Department of Mechanical Engineering, Manhattan College, Bronx, NY 18966, USA; ehsan.atefi@manhattan.edu; 3 Department of Psychology, Wake Forest University, Winston-Salem, NC 27109, USA; jayawide@wfu.edu; 4 School of Business, Stevens Institute of Technology, Hoboken, NJ 07030, USA; jqin2@stevens.edu; 5 Faculty of Engineering and Information Technology, University of Technology Sydney, Ultimo, NSW 2007, Australia; Amirhossein.Gandomi@uts.edu.au

**Keywords:** war, disaster, association rule learning, Sri Lanka, disaster survivors, war survivors, daily stressors, trauma, war trauma, machine learning

## Abstract

Research indicates that psychopathology in disaster survivors is a function of both experienced trauma and stressful life events. However, such studies are of limited utility to practitioners who are about to go into a new post-disaster setting as (1) most of them do not indicate which specific traumas and stressors are especially likely to lead to psychopathology; and (2) each disaster is characterized by its own unique traumas and stressors, which means that practitioners have to first collect their own data on common traumas, stressors and symptoms of psychopathology prior to planning any interventions. An easy-to-use and easy-to-interpret data analytical method that allows one to identify profiles of trauma and stressors that predict psychopathology would be of great utility to practitioners working in post-disaster contexts. We propose that association rule learning (ARL), a big data mining technique, is such a method. We demonstrate the technique by applying it to data from 337 survivors of the Sri Lankan civil war who completed the Penn/RESIST/Peradeniya War Problems Questionnaire (PRPWPQ), a comprehensive, culturally-valid measure of experienced trauma, stressful life events, anxiety and depression. ARL analysis revealed five profiles of traumas and stressors that predicted the presence of some anxiety, three profiles that predicted the presence of severe anxiety, four profiles that predicted the presence of some depression and five profiles that predicted the presence of severe depression. ARL allows one to identify context-specific associations between specific traumas, stressors and psychological distress, and can be of great utility to practitioners who wish to efficiently analyze data that they have collected, understand the output of that analysis, and use it to provide psychosocial aid to those who most need it in post-disaster settings.

## 1. Introduction

It is well established that individuals who are displaced by disaster and war—a figure currently at 70.8 million [1]—suffer from high rates of psychopathology. For example, in populations forcibly displaced by war, rates of depression range from 5.1% to 81% and rates of posttraumatic stress disorder (PTSD) range from 2.2% to 88.3% [2,3]. These mental health difficulties stem not only from their experience of trauma—for example, being bombed or being attacked [4,5]—but also from the constant life stressors that come up as a consequence of being in a disaster zone, such as lack of basic needs (e.g., food, water, shelter), lack of documentation needed for employment or travel, and limited options for employment [6,7,8,9]. Indeed, a considerable body of research now indicates that in disaster-affected populations, such stressors, along with traumatic experiences, play a significant role in the development of psychopathology. These stressors (1) are directly linked to post-disaster psychopathology and (2) partially, or on some occasions, completely mediate the relationship between experienced trauma and post-disaster psychopathology [10]. The strength of these findings is such that international aid organizations such as Medicins Sans Frontiers [11] and the Inter-Agency Standing Committee [12] have already noted in their guidelines that such life stressors should be the initial target of any post-disaster psychosocial intervention. 

Much of the research that has examined the relationship between stressful life events and psychopathology in disaster-affected populations typically collapse numerous daily stressors into a single composite variable [13,14,15,16] and then examine the relationship of this composite variable with experienced trauma and symptoms of psychopathology (variables that are often also represented by a single composite variable). Such an approach is necessary if one wants to use moderation and mediation models to examine the relationship between these variables; however, the results of such research is of limited use to practitioners who want to develop psychosocial interventions for a specific post-disaster context, as they do not specify which specific stressors are related to psychopathology and thus must be targeted. A number of recent studies have addressed this issue by employing big data mining techniques [17] such as network analysis, which allows one to create networks that visualize and describe relationships between variables and to identify relationships between specific traumatic events, stressors and symptoms of psychopathology [18,19,20]. This statistical approach allows one to identify key variables, whether they be stressors, traumatic events or symptoms of psychopathology that appear to be central to the network; in other words, their presence substantially increases the likelihood of the presence of the other symptoms and stressors. Practitioners can then identify those individuals who are struggling with those stressors or symptoms of psychopathology that are central to the network, since those individuals are more likely to be struggling with other problems.

Research that focuses on the role that life stressors play in the development of psychopathology in post-disaster settings has, without doubt, expanded our understanding of the key role these stressors play in the mental health of disaster survivors. However, the utility of such studies is limited for practitioners who are about to enter into a particular post-disaster setting with the goal of providing psychosocial help for those who are especially vulnerable to psychopathology. Given that the particular stressors in a post-disaster population vary from one particular disaster context to the next, it is unlikely that a research study that focuses on stressors in a different post-disaster population will be completely applicable to the current context. Furthermore, existing research on the role of stressors in post-disaster populations has thus far failed to identify a particular class of stressors that are especially impactful [10], so practitioners cannot count rely on the research literature to give them even a broad type of stressor to focus on. Instead, those providing interventions in a particular disaster-affected population often need to first gather data in order to identify the key stressors in that context [7]. 

Ideally, practitioners who have collected data on the salient stressors and traumas in a particular post-disaster population would be able to analyze it in such a way as to identify those stressors and traumas that are indicators of psychopathology. However, the statistical techniques that has thus far been used in the research literature to identify specific stressors and traumas, namely network analysis and cluster analysis, are complex and require the knowledge of advanced statistical methods—a resource that may not always be available to those working in these post-disaster populations. We propose that association rule learning (ARL), a big data statistical method [17,21] can be used as an alternative to network analysis and cluster analysis for the purpose of identifying profiles of traumas and stressful life events that predict psychopathology. Technically simpler than network analysis and easy to interpret and understand [22], this data mining technique identifies associations between discrete variables. ARL was created originally for market basket analysis, which is used by retailers to study the purchasing patterns of customers and specifically identify which items are likely to be bought together [22]. For example, one might discover, through ARL analysis, that customers who buy milk and butter are also more likely to buy bread. This rule can be depicted as:

{milk, butter}⟹{bread}

Here, milk and butter are considered the antecedent in the rule and bread is the consequent in the rule [21]. Two indices, support and confidence, are typically employed when selecting such rules [21]. Support refers to how often the antecedent occurs in the dataset. For example, if 40% of the customers in a dataset bought milk, butter, and bread, then the rule has a support of 40%. Confidence refers to how accurate the rule is, i.e., the ratio of the number of times the antecedent and consequent co-occur to the number of times that only the antecedent occurs. For example, if 85% of the customers who bought milk and butter also bought bread, then the confidence level for the rule that customers who buy milk and butter are also more likely to buy bread is 85%. 

The most commonly used method for identifying association rules in a dataset is the a priori algorithm [23], which identifies rules with a minimum support value and a minimum confidence value specified by users. When selecting these minimum values, one should aim for the highest possible values: if the minimum value is too low, the algorithm will create a large set of rules with low accuracy. Higher values lead to more meaningful, accurate rules. 

ARL generates easy-to-interpret rules that facilitate decision making, which differentiates this technique from other machine learning algorithms like cluster analysis and network analysis. Cluster analysis is used to investigate the existence of a relationship between variables [24] but does not generate explicit rules that ARL does, nor does it provide immediate information on the strength of the relationships. Network analysis is constructed from nodes and edges that represent the variables and connection between variables in the dataset. Defining the connection between the data points is user-dependent and very crucial in this algorithm [18,19,20]. For instance, one can define the connection (or edge) as the correlation between two data points. Then, the outcome of the network analysis becomes a very complex map showing the variables connected by edges representing their correlations. Interpreting each relationship between variables and the strength of the relationship is not as convenient as with ARL. Therefore, network analysis shows the existence of a relationship along with its strength, but it does not generate explicit rules. Furthermore, it should be noted that while network analysis has great promise as a method to identify networks of relationships between a wide range of variables of interest, the method is still in the process of being refined as a data analytical approach [25,26,27]. In particular, there are concerns that the indices that are calculated to measure the centrality of variables in the network are difficult to interpret [28], which suggests that such analyses would be of limited utility to practitioners in post-disaster settings who need a clear signal of which stressors and traumas are most indicative of psychological distress.

ARL has promise as an easy-to-use and easy-to-understand data analytic method that practitioners can use to efficiently analyze data they have collected in the post-disaster populations they hope to serve. In the current study, we aim to demonstrate the utility of ARL in identifying profiles of trauma and stressors that predict psychopathology by using the technique to identify such profiles in survivors of the Sri Lankan civil war [29]. Lasting from the period 1983 to 2009, this war was fought primarily between the Sri Lankan Armed Forces and the Tamil separatist group, the Liberation Tigers of Tamil Eelam (LTTE). At least 100,000 people died in the conflict, and a further 800,000 were displaced [30]. Those civilians caught in the midst of the conflict experienced numerous traumas—including rape, torture, shelling, aerial bombardment, forced recruitment into the LTTE—and life stressors—food and water shortages, loss of shelter, loss of employment and loss of material goods [31]. Ten years after the end of the war, over 42,000 internally displaced individuals still remain in Sri Lanka [1]. 

## 2. Method

### 2.1. Participants

Participants consisted of 337 Sri Lankan Tamil survivors of war receiving psychosocial services from the Family Rehabilitation Center (FRC), a Sri Lankan nongovernmental organization. Data were collected from FRC clinics in areas greatly impacted by war, namely the towns of Batticaloa, Jaffna, Nallur, Trincomalee, and Vavuniya. Data were collected over the period 2009–2011. Participants volunteered for the study and were paid 100 Sri Lankan rupees (approximately 75 U.S. cents in the period 2009–2011) in compensation. The average age of the participants was 43.41 years (*SD* = 13.7). Of the 337 participants, 185 participants were male (54.9%), 149 were female (44.2%) and three did not report their gender (0.9%). 

All study procedures were approved by the Institutional Review Board at the University of Pennsylvania, USA, and by the University of Peradeniya, Sri Lanka. 

### 2.2. Measures

Demographic Form—respondents completed a demographics form that included questions about their ethnicity, gender and age.

The Penn/RESIST/Peradeniya War Problems Questionnaire (PRPWPQ)—the PRPWPQ is a Tamil language questionnaire developed specifically for Sri Lankan war survivors that assesses a wide range of war-related traumas, stressors and symptoms of psychopathology [32,33]. The questionnaire was developed through the coding of 604 individual qualitative interviews conducted in North-Eastern Sri Lanka [31]. 

The PRPWPQ comprises three sections: (1) Trauma Exposure, (2) War-Related General Problems and (3) War-Related Psychological and Behavioral Problems (WRPBP). The Trauma Exposure section has two subsections—torture and other war trauma—and focuses on 22 different traumatic experiences (see Table 1). Respondents completing this section indicate whether they have experienced the trauma in question, and if so, indicate the number of times they had experienced that trauma. The War-Related General Problems section has five subsections: family problems (20 items), economic problems (10 items), social problems (26 items), lack of basic needs (9 items) and physical problems (19 items; see Table 2). Respondents indicate whether or not they have the problem in question by indicating either “yes” or “no.” The WRPBP section consists of culturally specific expressions of general anxiety, posttraumatic stress disorder, and depression symptoms (e.g., “Inability to make decisions [not knowing what to talk and what not to talk]”) as well as a few unique idioms of distress (e.g., “Not being able to work with a peaceful mind.). This section has three subscales: Anxiety, and two depression subscales titled Depression and Negative Perception. Respondents rate the severity of each symptom using the following scale: 1 (not at all); 2 (a little bit); 3 (quite a bit); 4 (extremely). Only the Anxiety and Depression subscales have been shown to predict functional impairment in this population [33], so only those two subscales were used in the current study. Cronbach’s alpha for the Anxiety and Depression subscales in the current study were α = 0.95 and α = 0.93 respectively.

## 3. Data Analysis

### 3.1. Conversion of WRPBP Items of the PRPWPQ into Discrete Variables

ARL analysis requires discrete variables; thus, we created discrete variables that indicated the presence of anxiety and depression as measured by the WRPBP. Ideally, one would use the clinical cut-offs determined for the measure being used to identify those individuals who are at risk for psychopathology. However, clinical cutoffs for WRPBP have not yet been developed, so we decided to calculate four discrete variables that indicated the presence of various levels of anxiety and depression: respondents who were experiencing at least some anxiety, respondents who were experiencing at least some depression, respondents who were experiencing severe anxiety, and respondents who were experiencing severe depression. We decided to calculate discrete variables that indicated the presence of some anxiety and depression since we assume that individuals who have some symptoms of anxiety and depression as indicated on a self-report questionnaire would warrant additional screening by a mental health professional; thus identifying those individuals through the use of profiles created by ARL would be of use. On the other hand, if resources for mental health interventions are scarce and practitioners only want to identify those individuals with severe anxiety and depression, they can use the variables that indicate the presence of more severe psychopathology. The four discrete variables created to measure various levels of anxiety and depression were as follows:

(1) **Some anxiety**: Respondents whose average score on the items in the anxiety subscale of the WRPBP section of the PRPWPQ was 2 (*a little bit*) or greater were considered to have at least some anxiety.
(2) **Severe anxiety**: Respondents whose average score on the items on the anxiety subscale of the WRPBP section of the PRPWPQ was 3 (*quite a bit*) or greater were considered to have severe anxiety.
(3) **Some depression**: Respondents whose average score on the items on the depression subscale of the WRPBP section of the PRPWPQ was 2 (*a little bit*) or greater were considered to have at least some depressive symptoms.
(4) **Severe depression**: Respondents whose average score on the items on the depression subscale of the WRPBP section of the PRPWPQ was 3 (*quite a bit*) or greater were considered to have severe depression.

### 3.2. Percentage of Individuals who Experienced Specific Traumas and Stressful Life Events and are Currently Experiencing Anxiety and Depression

We calculated the percentage of individuals who experienced specific trauma and stressful life events and are currently experiencing anxiety and depression (as defined in the section immediately above) as measured by the WRPBP section of the PRPWPQ. 

### 3.3. Identifying Association Rules

The Apriori algorithm [27] was used to identify those traumatic events and stressors that were associated with anxiety and depression. The binary set of variables $I=L\cup^{}R$ was constructed such that $L=\left\{L_{1},\ldots ,L_{106}\right\}$ represents the 106 traumas and stressors measured, and $R=\left\{R_{1},\ldots ,R_{4}\right\}$ represents the four variables measuring the presence of psychopathology. The binary dataset $D=\left\{D_{1},\ldots ,D_{337}\right\}$ containing 337 transactions was constructed based on the questioned answered by 337 respondents such that $D_{i}$ is a [1×110] vector that represented the data from the *i*th respondent. Each rule, LHS⟹RHS, that association rule algorithm finds shows that answering yes to the variables belonging to LHS (left-hand side, i.e., the antecedent) results in answering yes to a variable in RHS (right-hand side, i.e., the consequent). Rules are defined such that LHS⊆L and RHS⊆R, and all the rules must satisfy three thresholds:

(1) maximum rule length is 5;
(2) Support for the rules is larger than ϑ;
(3) Confidence for the rules is larger than ε.

Support was calculated as follows:

$$Support\;\left(LHS\right)=\frac{∥d\in D;LHS\subseteq d∥}{∥D∥}\geq \;\vartheta$$

where, ∥D∥=337 and ∥d∥ represents the number of respondents who answered yes to all the variables in LHS. Confidence was calculated as follows:

$$Confidence\;\left(LHS⟹RHS\right)=\frac{Support\;\left(LHS\cup RHS\right)}{Support\;\left(LHS\right)}\geq \varepsilon \;$$

We avoided creating redundent rules—i.e., complex rules containing a large number of variables on the *LHS* that nevertheless do not have a higher level of confidence than less complex rules (i.e., rules that have fewer variables in the *LHS*). If $LHS_{i}\subset LHS_{j}$ while $Confidence\;\left(LHS_{i}⟹RHS_{k}\right)\geq Confidence\;\left(LHS_{j}⟹RHS_{k}\right)$, $LHS_{j}$ is considered an unnecessary rule and was removed. This technique is used to modify the length of the LHS of the rules.

## 4. Results

### 4.1. Trauma Exposure

Table 1 presents the percentage of respondents who experienced specific traumas. Commonly experienced traumas included witnessing the injury of loved ones (47.5%), witnessing the death of loved ones (44.5%), being beaten in detention (36.5%) and being imprisoned (35.9%).

### 4.2. Stressful Life Events

Table 2 presents the percentage of respondents who experienced specific stressful life events. The vast majority of respondents indicated having economic problems, with all 10 items being endorsed by at least half of the sample, and seven of the 10 items being endorsed by more than 80% of the sample. Additionally, a majority of respondents indicated experiencing a lack of five out of the nine basic needs assessed (i.e., clothes, rights, medical help, food and security). Common family problems included insufficient support from relatives (48.7%), children/spouse having psychological problems (47.2%), not having a steady life because of duties towards family (46.6%) and being unable to take care of children (36.2%). Common social problems included fear of death (65.6%), problems being able to travel (57%), stress when moving to a new place (51.3%), not being able to do usual routines after having moved to a new place (49.9%), fear of being kidnapped (49.3%) and not being able to talk freely (46%). Common physical problems included body aches (62.6%), headaches (64.4%), backache (57.9%) and shivering (41.8%).

### 4.3. Anxiety and Depression

Table 3 shows the percentage of respondents who indicated that they experience some anxiety, severe anxiety, some depression and severe depression. A majority of the respondents experienced at least some anxiety and depression, but smaller percentages experienced severe anxiety (21.4%) and depression (15.1%).

### 4.4. Association Rules

**Figure 1:**
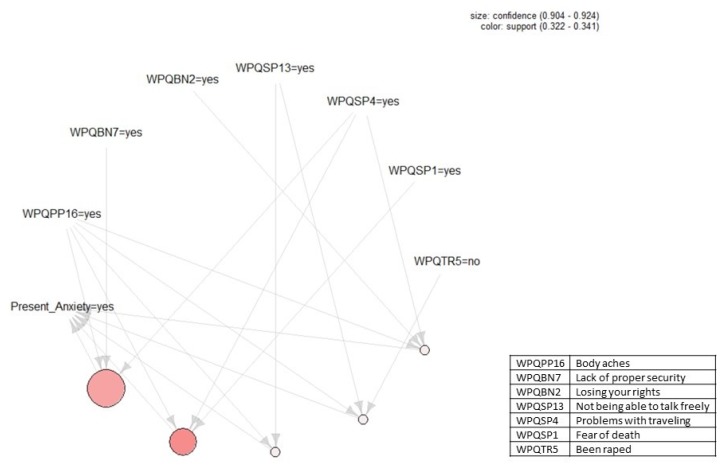
Association rules that identify those traumatic events and stressors that were associated with some anxiety. Threshold values for support and confidence are listed on the top right of the figure. Descriptions of the variables in the rules can be found in the box to the right.

**Figure 2:**
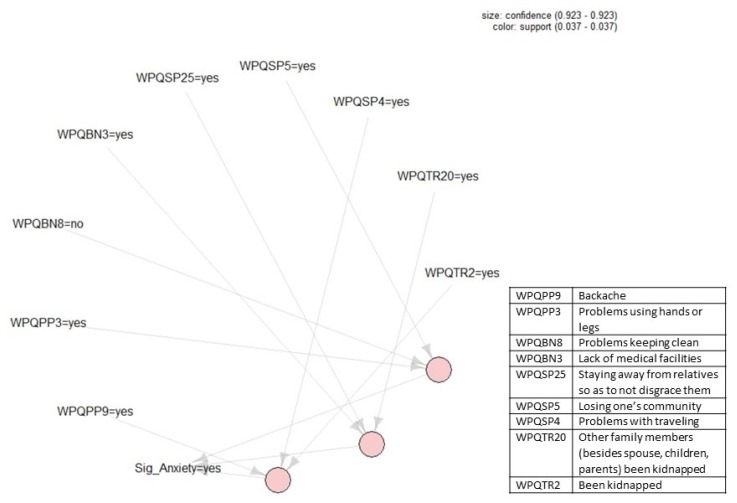
Association rules that identify those traumatic events and stressors that were associated with severe anxiety. Threshold values for support and confidence are listed on the top right of the figure. Descriptions of the variables in the rules can be found in the box to the right.

**Figure 3:**
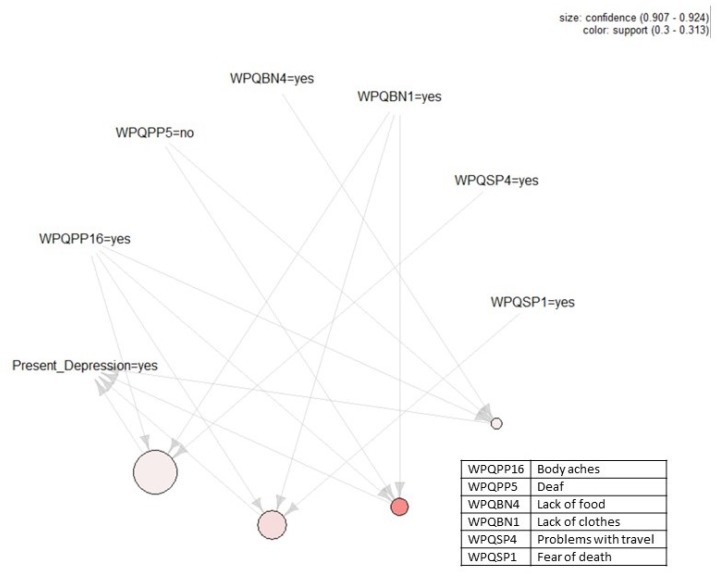
Association rules that identify those traumatic events and stressors that were associated with some depression. Threshold values for support and confidence are listed on the top right of the figure. Descriptions of the variables in the rules can be found in the box to the right.

**Figure 4:**
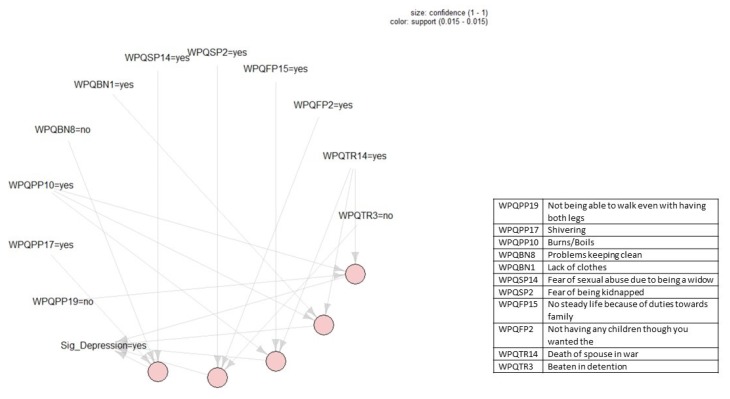
Association rules that identify those traumatic events and stressors that were associated with severe depression. Threshold values for support and confidence are listed on the top right of the figure. Descriptions of the variables in the rules can be found in the box to the right.

Figure 1, Figure 2, Figure 3 and Figure 4 present the association rules that identify traumatic events and stressors that are associated with anxiety and depression. These rules are represented by circles at the bottom of each figure. Each circle represents the *LHS* of a rule and arrows show the *RHS*. Threshold values for support and confidence are listed on the top right of each figure.

Figure 1 shows the five association rules that identify those traumatic events and stressors that were associated with some anxiety in the current dataset. Moving from Figure 1’s left-hand side to the right-hand side, the rules are:

(1) {Body aches, Problems with travel, Lack of proper security}⟹{Some anxiety}

(2) {Body aches, Problems with travel, Fear of death}⟹{Some anxiety}

(3) {Body aches, Not being able to talk freely}⟹{Some anxiety}

(4) {Body aches, Not being able to talk freely, Not being raped}⟹{Some anxiety}

(5) {Body aches, Losing your rights, Problems with travel}⟹{Some anxiety}

Two threshold values of ϑ=0.3 and ε=0.9 were set to construct these rules for support and confidence, respectively. This indicates that all rules have confidence of at least 90% and support of at least 30%. For example, for the rule presented in Equation (1) above, 30% of respondents (112 people) answered yes to all four variables of I = *{Problems with travel, Lack of proper security, Body aches, Some anxiety}.* In addition, 90% of respondents (100 people) that answered yes to all the variables in *LHS = {Problems with travel, Body aches, Lack of proper security}* also answered yes to *RHS = {Some anxiety}.* The threshold levels of ϑ and ε are mainly related to the dataset size and the number of respondents who answered yes to the *R*HS. Considering the current dataset’s size, we selected threshold values such that we can have as large a possible number of respondents for each rule to generate strong rules with large confidence. Lowering the level of support results in generating rules that sometimes are redundant and supported by a small number of transactions, whereas selecting a higher support threshold might result in not generating any rules. We decided to start from the support threshold of ϑ=0.3, which refers to 112 respondents who answered yes to the variables constructing each rule and to decrease the threshold if needed.

The size of each circle in Figure 1 shows the confidence level. Thus, the largest circle has the confidence level of 0.92, and the smallest circle has the confidence level of 0.9. Each circle’s color indicates the support level. Hence, circles with a darker color have higher support closer to 0.34, and circles with a lighter color have lower support closer to 0.32.

Figure 2 shows three association rules that identify those traumatic events and stressors that were associated with severe anxiety in the current dataset. Two threshold values of ϑ=0.03 and ε=0.9 were set to construct these rules for support and confidence, respectively. Note that the threshold value for support here is much lower (0.03) than the one we used to identify those stressors and traumas that predicted some anxiety (0.3), because only 20% of respondents indicated they had severe anxiety. Figure 2 shows that all three rules had the same confidence level as indicated by the similar size of each of the circles representing the rules (ε=0.92), as well as the same support level as indicated by the shade of the circle (ϑ=0.04). Moving from Figure 2’s left-hand side to the right-hand side, the three identified association rules are:

(6) {Backache, Problems with travel, Been kidnapped}⟹{Severe anxiety}

(7) $$\left\{\begin{array}{c} Lack\;of\;medical\;facilities,\;\;Staying\;away\;from\;relatives\;so\;not\;to\;disgrace\;them,\;Family\;\\ members\;\left(besides\;spouse,\;children,\;parents\right)\;been\;kidnapped\; \end{array}\right\}⟹\;\left\{Severe\;anxiety\right\}$$

(8) $$\left\{Problems\;using\;hands\;or\;legs,\;Problems\;keeping\;clean,\;Losing\;one^{\prime }s\;community\;\right\}⟹\;\left\{Severe\;anxiety\right\}$$

Figure 3 shows our association rules that identify those traumatic events and stressors that were associated with some depression in the current dataset. Two threshold values of ϑ=0.3 and ε=0.9 were set to construct these rules, as with some anxiety, for support and confidence, respectively. The largest circle in Figure 3 has the confidence level of 0.92, and its smallest circle has the confidence level of 0.91. Circles with a darker color have higher support closer to 0.31 and circles with a lighter color have lower support closer to 0.3. Moving from Figure 3’s left-hand side to the right-hand side, the four identified association rules are:

(9) {Body aches, Lack of clothes, Problems with travel}⟹{Some depression}

(10) {Body aches, Lack of clothes, Fear of death}⟹{Some depression}

(11) {Body aches, Lack of clothes, Not being deaf }⟹{Some depression}

(12) {Body aches, Not being deaf, Lack of food}⟹{Some depression}

Lastly, five association rules that identify those traumatic events and stressors that were associated with some depression in the current dataset can be found in Figure 4. For constructing these rules, we set a threshold value for the confidence of ε=0.9. However, the threshold value for support had to be set at ϑ=0.01 given that only little over 15% of respondents indicated that they had severe depression. As can be seen in Figure 4, all five rules had the same confidence level as indicated by the similar size of each of the circles representing the rules (ε=1) as well as the same support level as indicated by the shade of the circle (ϑ=0.15). Moving from Figure 4’s left-hand side to the right-hand side, the five identified association rules are:

(13) {Shivering, Not having problems keeping clean, Fear of sexual abuse due to being a widow}⟹{Severe depression}

(14) $$\left\{\begin{array}{c} Fear\;of\;being\;kidnapped,\;Not\;having\;children\;though\;you\;wanted\;them,Not\;been\\ beaten\;in\;detention \end{array}\right\}⟹\;\left\{Severe\;depression\right\}$$

(15) $$\left\{\frac{Burns}{boils},No\;steady\;life\;because\;of\;duties\;towards\;family,\;Death\;of\;spouse\;in\;war\;\right\}⟹\;\left\{Severe\;depression\right\}$$

(16) $$\left\{\frac{Burns}{Boils},\;Lack\;of\;clothes,\;Death\;of\;spouse\;in\;war\right\}⟹\left\{Severe\;depression\right\}$$

(17) $$\left\{\frac{Burns}{Boils},\;Not\;having\;problems\;walking\;with\;both\;legs,\;Death\;of\;spouse\;in\;war\right\}⟹\;\left\{Severe\;depression\right\}$$

## 5. Discussion

In this paper, we demonstrated how ARL can be used to identify profiles of trauma and stressors that predict anxiety and depression in a sample of Sri Lankan war survivors. We identified five trauma-and-stressor profiles that predicted the presence of some anxiety, three profiles that predicted the presence of severe anxiety, four profiles that predicted the presence of some depression, and five profiles that predicted the presence of severe depression. Practitioners working in post-disaster settings can use profiles such as these to identify those individuals at risk for psychopathology and then provide them with any needed interventions. For example, if a practitioner working with Sri Lankan war survivors wants to target individuals at risk for severe anxiety, she can use the three association rules for Severe anxiety Equations (6)–(8) to identify those survivors. For example, the practitioner may want to identify those survivors who experience body aches, problems with travel and have been kidnapped, the stressors that comprise the antecedent in Equation (6) and have them come in for further evaluation.

The repeated presence of one or two particular variables in the antecedent of several rules (for example, the presence of Body Aches and Lack of Clothes in the antecedent in Rules 9–12) points to the fact that these variables are dominant in predicting the occurrence of anxiety and depression. However, in order to reach a high confidence level and thus have a more accurate rule, we need the presence of a third variable in the antecedent of the rule. To draw an example from our analyses: the variables Body aches and Lack of clothes are present in the antecedent in Rules 9-12. If we remove the third variable from the antecedent of these rules, the confidence of each rule decreases by 3% to 7%. One can reduce the confidence to lower the complexity and keep only Body aches and Lack of clothes in the antecedent of the rules. However, to keep confidence above 90% and thus have more accurate rules, all three variables are required. 

There are two key reasons why we believe ARL is especially useful to practitioners working in post-disaster settings. First, as we stated earlier, and hopefully have demonstrated, this technique—compared to other statistical methods-is simple to understand and its results are easy to interpret [22]. A user-friendly, data-analytic online application could be created where practitioners could upload their data to; users can then adjust the support and confidence threshold criteria (which will be set at reasonable default thresholds) and then analyze their data to identify association rules. Given the simplicity of the algorithm used to identify association rules and the ease with which one can interpret its output, such an application would not run into the issues faced by black-box machine learning applications developed to address other social problems, such as criminal justice sentencing and medical diagnosing, the results of which are often uninterpretable [34]. We hope to create such an application in the near future.

The second reason why ARL is of considerable utility to practitioners working in post-disaster populations is that one can use the technique even in small datasets and obtain results that can potentially be of use. Only 51 out of 337 respondents had severe depression in our demonstration of the technique here; yet we were able to identify five profiles of traumas and stressors that predicted the presence of severe depression. The fact that one can adjust the threshold for support means that one can identify rules in relatively small datasets. As we noted previously, there are limitations to lowering the support threshold, i.e., generating a large set of rules with low accuracy. Nevertheless, ARL allows practitioners to come up with actionable analyses using whatever data they have been able to collect. Association rules developed using small datasets will indeed be less accurate that those developed using larger datasets; yet the method allows its users to maximize the knowledge gained for the data they have collected. 

We believe that ARL would be especially useful in post-disaster settings in low-and-middle-income countries, where there are often only limited resources for psychosocial interventions and where between 76–85% of individuals with severe mental illness receive no treatment in low-and-middle-income countries mainly due to lack of resources [35]. Practitioners can use data collected on symptoms of psychopathology, stressors and traumas from a sample of survivors to identify—using ARL-a set of stressors and traumas that, when experienced together, predict increased risk for psychopathology. Practitioners can then use that information to identify those survivors who require more extensive psychological evaluation. Once practitioners know what traumas and stressors are associated with higher risk of psychopathology, they can send out short surveys that ask respondents if they have experienced or are currently experiencing those traumas and stressors that are predictive of psychopathology. Such short surveys could be distributed electronically via internet or mobile technologies, which have been identified as playing a key future role in expanding assessment and treatment of mental illness in post-disaster low-and-middle-income countries [36]. Given their brevity, one would expect to see high rates of response to these surveys. In addition, in contexts where there is a stigma attached to mental illness, response rates for these short surveys may be higher because the surveys avoid asking questions on psychopathology from respondents. Furthermore, if questions regarding trauma (for example, rape) are deemed as too stigmatizing [37], the flexibility of ARL is such that you can create profiles consisting solely of stressors.

We want to acknowledge that ARL can be a key addition to the toolbox of those practitioners who work in post-disaster contexts, but its utility is limited to identifying traumas and stressors that tend to co-occur with psychopathology. ARL is an unsupervised learning method that creates descriptive models of patterns in datasets [17]. Such methods are typically exploratory and data-driven. We emphasize that the utility of ARL lies in its ability to enable practitioners working in disaster settings to identify collections of stressors and traumas that are indicative of psychopathology. However, this technique is of limited use to those researchers who aim to better understand the nature of the relationship between symptoms of psychopathology, stressors and traumas-other statistical techniques such as cluster analysis and network analysis are more suited to answer such questions. Network analysis allows users, for example, to create networks of partial correlations between variables, i.e., correlations that are statistically controlled for all other associations in the network [18,19,20]. Such networks can identify potentially causal relationships between variables. Researchers should continue to use network analyses and other appropriate data analytical approaches to uncover the relationship between trauma, stressors and psychopathology in post-disaster settings.

## 6. Conclusions

We believe that ARL has a place in post-disaster psychosocial intervention work. Given that budgets for post-disaster intervention are often insufficient to disaster survivors’ needs, ensuring that field workers have access to these resources is key to them having the most impact on those who are in most need. Techniques such as ARL allow practitioners to identify those who are at most risk for psychopathology.

## Figures and Tables

**Table 1:** Experienced Trauma as assessed by the Trauma Exposure section of the Penn/RESIST/Peradeniya War Problems Questionnaire (n = 337).

Percentage	%
Witnessed the injury of loved ones	47.5
Witnessed the death of loved ones	44.5
Beaten in detention	36.5
Been imprisoned	35.9
Family members (besides spouse, children, parents) been kidnapped	17.5
Injured by airstrikes or bomb explosions or sudden attacks	15.4
Been kidnapped	12.5
Tortured by being beaten with a bag containing petrol	11.9
Children been kidnapped	11.6
Death of child/children in war	11.3
Death of spouse in war	9.2
Children been handicapped	9.2
Tortured by being pricked under the nail with a pin	9.2
Death of mother and/or father in war	8.9
Tortured by being burnt with a cigarette butt in detention	8.6
Electrocuted in detention	7.7
Husband or wife been kidnapped	7.7
Husband or wife been handicapped	5.6
Tortured by being forcibly fed mosquito coil	4.2
Caught in a land mine	3.3
Parent(s) been kidnapped	2.4
Been raped	1.2

**Table 2:** War-Related General Problems as assessed by the War-Related General Problems section of the Penn/RESIST/Peradeniya War Problems Questionnaire (n = 337).

Percentage	%
**Family Problems**	
Insufficient support from relatives	48.7
Children/spouse has psychological problems	47.2
No steady life because of duties towards family	46.6
Unable to take care of children	36.2
Having been separated from husband/wife/children/other relatives	28.5
Unable to get children married/give dowry	26.7
Not being able to travel to meet relatives due to travel restrictions	26.4
Not having anyone to take care of you in old age	23.7
Being dependent on relatives	22.6
Problems with husband/wife at home	22.6
Taking care of your children and siblings as a single person	20.5
Problems between children	20.2
Unable to control (i.e., discipline) your children	18.4
Problems with marriage plans	11
Alcohol abuse by self	9.2
Alcohol abuse by husband or wife	7.4
Not having any children though you wanted them	7.4
Being dependent on wife	7.4
Alcohol abuse by parents	4.2
Not being properly looked after or cared for by children	0.6
**Economic Problems**	
Not being able to earn enough money for your basic needs	89.3
Not having money	86.1
Loss of material goods	84.9
Unavailability of employment	83.1
Not being able to work due to illness	83.1
Financial debt	82.8
Not being able to do the job you desire	81
Loss of house/land	77.2
Loss of work equipment	73
Not beingable to work due to being a single parent	52.2
**Social Problems**	
Fear of death (from bombs/ land mines/armed groups)	65.6
Problems with travel	57
Stress when moving to a new place	51.3
Not being able to do usual routines after having moved to a new place	49.9
Fear of being kidnapped	49.3
Not being able to talk freely	46
Not having offical documents	33.5
Losing one’s community	30
Living with relatives	25.5
Unable to participate in cultural events	21.1
Living alone (without anyone)	20.8
Having to give bribes to get basic services	16.9
Not being respected by society	16.6
Lack of security due to being alone	16.3
Staying away from relatives so not to disgrace them	16.3
Isolated in society due to unemployment	13.1
Been a victim of theft	12.2
Isolated in society due to history of being imprisoned	11.9
Living in a camp	11
Neglected by society	10.7
Living with non-relatives	9.8
Not being able to get married	7.4
Isolated from society due to being a widow	7.1
Problems with neighors or others in the camp	6.2
Fear of sexual abuse due to being a widow	5.9
Unable to get married due to stigma	3.3
**Lack of Basic Needs**	
Lack of proper security	62.3
Losing your rights	59.9
Lack of medical facilities	59.9
Lack of food	54.9
Lack of clothes	53.4
Lack of fuel	49
Not being able to obtain education	40.1
Lack of water	34.4
Problems keeping clean	30.9
**Physical Problems**	
Body aches	62.6
Headaches	64.4
Backache	57.9
Shivering	41.8
Eye problems	33.5
High blood pressure	22
Problems using hands or legs	21.1
Loss of teeth	17.8
Not being able to walk even with having both legs	16
Heart problems	15.7
Burns/boils	15.4
Fractures	14.8
Deaf	10.1
Head injury	10.1
Retention of bullet or bomb particles in the body	9.5
Loss of arms or legs in a landmine	6.2
Kidney problems	7.1
Stroke/blood clots	6.8
Loss of arms or legs of a child or spouse or breadwinner due to a landmine	4.2

**Table 3:** Percentage of respondents who have anxiety and depression as assessed by the Anxiety and Depression subscales of the Penn/RESIST/Peradeniya War Problems Questionnaire (n = 337).

Percentage	%
Some Anxiety	67.1
Severe Anxiety	21.4
Some Depression	63.8
Severe Depression	15.1
